# Glucocorticoid-Induced Bone Loss Is Associated with Abnormal Intravertebral Areal Bone Mineral Density Distribution

**DOI:** 10.1155/2013/768579

**Published:** 2013-05-08

**Authors:** Louise I. Manning, Andrew M. Briggs, Sharon Van Doornum, Ashwini Kale, Susan Kantor, John D. Wark

**Affiliations:** ^1^University of Melbourne, Department of Medicine, Royal Melbourne Hospital, Parkville, VIC 3050, Australia; ^2^Bone and Mineral Service, Royal Melbourne Hospital, Parkville, VIC 3050, Australia; ^3^Curtin Health Innovation Research Institute, Curtin University, Bentley, WA 6102, Australia; ^4^Arthritis Victoria and Osteoporosis Victoria, Elsternwick, VIC 3185, Australia

## Abstract

Individuals with glucocorticoid-induced osteoporosis experience vertebral fractures at an increased rate and at higher vertebral areal bone mineral density (aBMD) than individuals with primary osteoporosis. Standard posteroanterior- (PA-) projection dual energy X-ray absorptiometry (DXA) lacks the diagnostic sensitivity required for reliable estimation of vertebral fracture risk in individuals. Assessment of subregional vertebral aBMD using lateral-projection DXA may improve the predictive value of DXA parameters for fracture. One hundred and four individuals were recruited and grouped for this study: primary osteoporosis with no history of vertebral fracture (*n* = 43), glucocorticoid-induced bone loss (*n* = 13), and healthy controls (*n* = 48). Standard PA-projection and supine-lateral scans were performed, and lateral scans were analysed according to an established protocol to measure aBMD within 6 subregions. Main effects for subregion and group were assessed and observed, by ANCOVA. Ratios were calculated between subregions and compared between groups, to overcome the potentially confounding influence of variability in subregional geometry. Significantly lower values were observed in the glucocorticoid group for the ratios of (i) anterior subregion: whole vertebral body and (ii) posterior: whole vertebral body when compared to the primary osteoporosis and control groups (*P* < 0.05). Lower anterior subregional aBMD in individuals on glucocorticoid therapy may help to explain the increased vertebral fracture risk in this patient group.

## 1. Introduction

Glucocorticoid therapy is widely used for the management of inflammatory and allergic conditions. While being effective in ameliorating these conditions, glucocorticoids can adversely affect bone quality and bone strength [1], thereby leading to an increased propensity to fracture. Secondary bone fragility or osteoporosis due to glucocorticoid therapy, termed glucocorticoid-induced osteoporosis (GIO), is a clinically important phenomenon. This is primarily because of reduced skeletal integrity predisposing to fragility fracture, but also because patients with GIO experience vertebral fractures at a rate which exceeds individuals with primary osteoporosis, despite a comparable areal BMD (aBMD) [2–7]. Identifying possible mechanisms underlying this observation is therefore important. 

The effects of glucocorticoids are mediated by the cytosolic glucocorticoid receptor [8] (cGCR), which is expressed on a variety of skeletal and extraskeletal cells [9]. The adverse effects of glucocorticoids on bone are complex and are both direct and indirect. Important direct effects include an early transient increase in osteoclastic bone resorption, a reduction in osteoblast differentiation and function, and an increase in osteocyte apoptosis [10]. There may also be indirect effects leading to increased fracture risk including glucocorticoid-induced myopathy [11].

This may explain the rapid early phase of bone loss upon commencement of therapy. Glucocorticoids similarly affect bone loss due to their proapoptotic effects on osteoblasts and osteocytes [8, 12, 13]. Defects in the osteocyte network within bone negatively affect bone mineralisation and microarchitecture and may explain the exaggerated trabecular bone loss [3, 7, 14]. 

Vertebral fractures are the most common type of osteoporosis-related fracture [15–17] and often present without obvious acute symptoms [18], leading to lack of recognition and treatment. Vertebral fractures frequently occur spontaneously or from minor trauma [18] and are associated with morbidity including decreased physical function (e.g., balance and muscle function), loss of height, compromised pulmonary capacity, increased thoracic kyphosis, and acute and chronic back pain [6, 15, 17–21]. These sequelae become more significant with increasing numbers of vertebral fractures. Vertebral fractures strongly predict the risk of future fracture [22–25], in particular subsequent vertebral and, to a lesser extent, appendicular fractures. 

Possible explanations for the increased vertebral fracture risk among individuals with GIO compared to those with primary osteoporosis may lie in the pathophysiology of bone loss with underlying systemic inflammatory disease in these individuals, in particular rheumatoid arthritis, which has been found to be associated with increased fracture risk [26, 27], the pharmacokinetics of glucocorticoids, or perhaps poor measurement specificity of bone parameters like aBMD in these individuals. 

Posteroanterior- (PA-) projection dual energy X-ray absorptiometry (DXA) is the most commonly used modality for the measurement of aBMD at the hip and lumbar spine [28]. A strong relationship exists between aBMD and bone mineral content (BMC) derived from PA-projection DXA scans [29] and these variables are used ubiquitously to diagnose and monitor skeletal integrity. However, they cannot be used to reliably predict an individual's vertebral fracture risk, particularly in the context of glucocorticoid-induced bone loss [30–32]. Lateral-projection DXA has better specificity and sensitivity when detecting vertebral osteoporosis and age-related bone loss and is better able to detect vertebral fractures than PA-projection scanning [30, 32–35]. These attributes of lateral-projection scanning are most likely related to the ability to isolate the vertebral body from the cortical bone-rich posterior elements of the vertebra, thus limiting the analysis to the metabolically-active trabecular bone of interest. 

Lateral-projection scans also enable measurement of BMC and aBMD in intravertebral subregions. Variation in aBMD between intravertebral subregions could explain why one individual may sustain a vertebral fracture whilst another may not, due to subregional variation in bone compressive strength; in essence, a chain is only as strong as its weakest link. *Ex vivo* research undertaken by Wegrzyn et al. [36] supported this hypothesis and suggested that trabecular microarchitecture and its associated regional heterogeneity contribute to vertebral fracture risk prediction. Older studies utilising histomorphometry, QCT, and DXA techniques have consistently shown relatively lower bone volume and trabecular thickness in both central and anterior vertebral subregions [36–40]. These *ex vivo* studies suggest that there could be a difference in intravertebral aBMD between individuals with and without vertebral fracture and potentially in those with glucocorticoid-induced bone loss. 

A technique to measure *in vivo* vertebral subregional aBMD has been developed by our research team with a matched supine-lateral DXA scanning technique using a Hologic QDR4500A densitometer [41]. Subsequent studies have further validated the subregional methodology, establishing high short-term *in vivo* precision [41], moderate to high intrarater and interrater precision [41], and concurrent validity when compared with pQCT and micro-CT [42]. Validation findings were later replicated using a much larger sample size [43], demonstrating that lateral DXA is valid when measuring heterogeneity in aBMD between intravertebral subregions. Lateral DXA measurement of aBMD has also been shown to be a better predictor of vertebral failure load at both the whole vertebral body [44] and within subregions [45], compared with standard PA DXA. 

BMC derived from DXA is directly influenced by bone size; thus, comparing BMC between large and small stature people becomes problematic. While normalising BMC to projection area, creating aBMD (g/cm^2^), corrects somewhat for variability in bone size, aBMD is still influenced by bone size as it cannot account for bone depth and thus cannot measure true (volumetric) bone density, which remains independent of bone size. Consequently, comparing subregional aBMD (srBMD) between individuals is not valid, due to inevitable heterogeneity in subregional bone geometry among individuals. To minimise the potentially confounding effect of variable subregional bone geometry on srBMD differences, ratios of srBMD may be compared between individuals. Deriving a ratio index of srBMD from an individual and comparing it to the same ratio in other individuals eliminate the potentially confounding effect of differences in subregional geometry between individuals. Therefore, the aim of this study was to compare srBMD ratios between individuals with primary and secondary osteoporosis as a means to explain a possible mechanism underlying the increased rate of vertebral fractures observed in GIO populations. 

## 2. Materials and Methods

### 2.1. Design

A cross-sectional cohort study was undertaken in a tertiary hospital-based bone densitometry unit (BDU).

### 2.2. Participants

A convenience sample of 104 individuals was recruited sequentially for this study to form three participant groups. These were (i) individuals diagnosed with primary osteoporosis who had no history of vertebral fracture (*n* = 43), (ii) individuals diagnosed with glucocorticoid-induced osteoporosis (GIO) or glucocorticoid-induced osteopenia who had no history of vertebral fracture (*n* = 13), and (iii) healthy controls (*n* = 48). Participants in the GIO and primary osteoporosis groups were referred to the BDU for clinical scanning purposes, while control participants were recruited from the community through advertisements in local media. The study protocol was approved by the Human Research Ethics Committee of Melbourne Health (2009.085). All participants provided written, informed consent to participate.

General inclusion criteria were that all participants were required to be aged ≥50 years old; female participants had to have been at least 5-year postmenopausal, defined as 5 years or more, since their last menstrual period; and all individuals were required to be independent with ambulation and activities of daily living. General exclusion criteria included a history of spinal surgery, any vertebral fracture classified as Grade 1 or higher according to the Genant criteria [46], a body mass index (BMI) greater than 33 or less than 18 kg/m^2^, any formally diagnosed musculoskeletal conditions in the spine that may affect the accuracy of a spinal DXA scan, and the current use of bone-active therapies. To be included in the glucocorticoid group, participants needed to have had a history of glucocorticoid use of ≥5 mg/day of prednisolone continuously for at least 6 months within the last 3 years along with the diagnosis of either osteoporosis or osteopenia, based on WHO criteria, using DXA-derived *T*-scores (*T*-score ≤ −1.5) from either the total hip, total spine, or L3 vertebral body. Individuals in the primary osteoporosis group were required to have a diagnosis of osteoporosis based on WHO criteria (*T*-score ≤ −2.5) at the total hip, total spine, or L3 vertebral body from the PA-projection DXA scan. Individuals in the control group were required to have normal BMD based on WHO criteria (*T*-score > −1.0) at all of these sites. Eligibility to participate was determined from a telephone interview and a review of DXA data from formal clinical scans (GIO and primary osteoporosis groups) or voluntary research scans (control group). 

### 2.3. Protocol

Each participant attended the BDU where demographic and clinical data were collected via questionnaire and height (cm) and mass (kg) measured to ensure their BMI fell within the required range of 18–33 kg/m^2^. After DXA scanning, a 10-year risk of sustaining a hip or major osteoporotic fracture was calculated using the FRAX tool using Australian reference data [47].

### 2.4. Bone Densitometry

#### 2.4.1. DXA Scanning

All participants were scanned on a Hologic QDR4500A fan beam densitometer (Bedford, MA, USA), using Hologic software version 9.10D. To monitor the reproducibility of the machine's results, a quality control spine phantom with known BMC was scanned daily prior to any patient scans. Over the data collection period, the mean coefficient of variation (%CV) was 0.35%, indicating excellent temporal stability of the machine. 

All participants underwent standard PA-projection scanning of the hip and spine, following standard Hologic protocols to derive aBMD, BMC, *T*-scores, and *Z*-scores at the right hip and spine, either for clinical (GIO and primary osteoporosis groups) or research (control group) purposes. These standard scans were used to determine group eligibility according to WHO classification criteria. In order to exclude the presence of vertebral fracture, Instant Vertebral Assessment (IVA) scans were acquired to assess vertebral morphometry between T5 and L4 (Hologic software 9.10D). IVA has been reported to have high specificity (>90%), high sensitivity (70–86%), negative predictive values (93.6–99.4%), and good reliability between raters [16, 17, 25, 48, 49]. A matched supine-lateral scan of the lumbar spine was then acquired using the array scanning mode and this scan set was used for measurement of srBMD. 

#### 2.4.2. DXA Analysis

Standard PA DXA scans of the hip and spine were analysed according to the manufacturer's instructions [50]. A combination of qualitative and quantitative methods was used to identify and diagnose vertebral fracture(s) on the basis of images gained from IVA scanning. Vertebral fractures were defined as grade 2 deformity or higher according to the semiquantitative method described by Genant et al. [46]; a method with excellent reproducibility [51, 52]. This was achieved by visual inspection of vertebral morphometry, from the IVA scan, and a measured reduction in anterior, middle, or posterior vertebral height of at least 25–40% or a 20–40% reduction in vertebral area, as calculated by Hologic software 9.10D. Adjustment factors calculated from normal vertebral height ratios, established by Diacinti et al. [53] for T5-L4, were included in the calculation of height ratios, serving as reference data to limit overestimation of fracture incidence. The reliability of IVA scans to diagnose vertebral fractures has been established previously by Chapurlat et al. [49]. 

**Figure 1:**
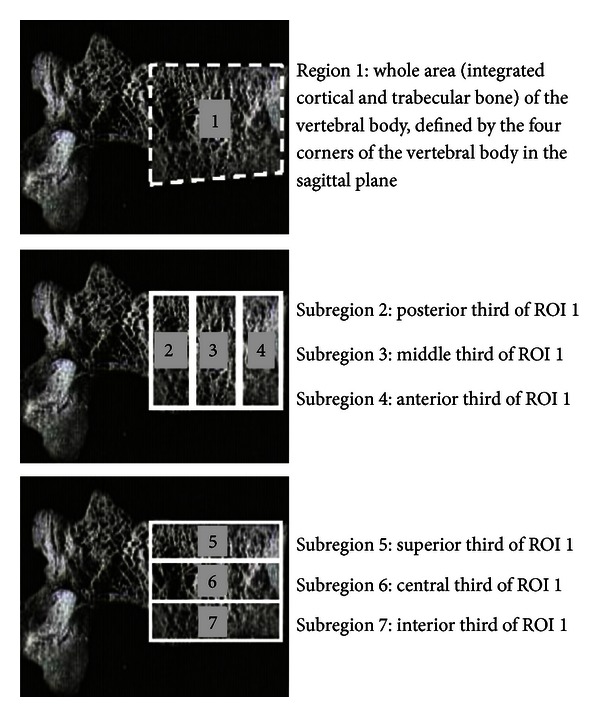
DXA-derived vertebral subregions defined using Hologic software. ROI 1 (whole) was defined by the four corners of the vertebra. ROIs 2–4 (posterior, middle, and anterior) formed equal thirds in the area of ROI 1, oriented sagittally. ROIs 5–7 (superior, central, and inferior) formed equal thirds in area of ROI 1, oriented transversely. Reprinted from Briggs et al. [42] with permission from Elsevier Copyright Clearance Center.

Lumbar spine images acquired from the lateral DXA scans were used to calculate aBMD in seven regions of interest (ROIs) at the L3 vertebral body (Figure 1), consistent with established protocols for which reliability and validity have been established [38, 41–45, 54]. L3 was used as the target vertebra as it is less affected by overlapping ribs than L2 [55]. Prior to the subregional ROI analysis, the global ROI window used during the Hologic analysis was set with a maximum height of 151 pixels and width of 141 pixels to ensure consistent software-defined analysis parameters between images. ROI 1 was defined as the entire vertebral body area and hence was demarcated by the four borders of the L3 vertebral body (superior and inferior endplates and anterior/posterior edges of the centrum). ROI 1 was therefore comparable to the vertebral area used to calculate aBMD in the standard Hologic analysis of lateral scan data. ROIs 2–7 consisted of six intravertebral subregions, of which the size and shape were selected manually. ROIs 2–4 were sagittally orientated, equally dividing ROI 1, by width, into thirds. ROIs 5–7 were oriented transversely, again dividing the total area of ROI 1 into equal thirds, by height. A consistent pixel width and height for sagittal and transverse subregions were employed to maintain the uniformity of dimensions. After the size and shape of the subregions were defined, individual aBMD measurements were calculated for each ROI.

### 2.5. Data Analysis

Descriptive and clinical characteristics were compared between the three groups using a one-way ANOVA. Differences in srBMD between groups were examined with two 3 × 4 ANCOVAs with one repeated measure. For each model, “group” was set as the between-subject factor (*k* = 3) and ROI as the within-subject repeated measure (*k* = 4) *a priori*, while L3 vertebral body area (ROI 1 area), derived from the lateral-projection scan, was used as a covariate to account for observed variability in bone size between the groups. To ensure that no overlapping subregions were compared *post hoc*, ANCOVA 1 included whole vertebral area (ROI 1) and the three subregions orientated sagittally (ROIs 2–4), whilst ANCOVA 2 included ROI 1 and the three subregions orientated transversely (ROIs 5–7). 

To minimise the potentially confounding influence of subregional geometry on subregional BMD values between groups, ratios of srBMD were compared between groups with a one-way ANOVA. Twelve ratios were derived: six comparing subregional values (ROIs 2–7) to the whole vertebral area (ROI 1) and six comparing non-overlapping subregions, consistent with an earlier analysis [54]. 

The critical alpha level of significance was set at 0.05 (2-tailed) and Bonferroni corrections were made for multiple *post hoc* comparisons in all statistical models. Statistical analysis was undertaken using SPSS version 19.1 for Windows.

## 3. Results

### 3.1. Descriptive Measures

While there was no difference in age and height between the groups, there was a significantly greater proportion of males in the GIO group compared to the other groups (Table 1). Significant differences were also observed between the groups for mass, BMI, FRAX score, *T*-scores for the spine and hip, and L3 vertebral body area (ROI 1). The GIO group had significantly higher mass (mean difference (MD) = 13.77 kg, 95% CI = 4.96–22.58), BMI (MD = 3.00 kg/m^2^, 95% CI = 0.48–5.53), *T*-scores at the PA total spine (MD = 0.99 SD, 95% CI = 0.16–1.82) and the total hip (MD = 0.69 SD, 95% CI = 0.06–1.32), and a significantly larger L3 vertebral body area (MD = 2.27 cm^2^, 95% CI = 0.46–4.07) compared to the primary osteoporosis group. When compared to controls, the primary osteoporosis group was observed to have significantly lower mass (MD = 10.06 kg, 95% CI = 4.33–15.91), lower BMI (MD = 2.65, 95% CI = 0.97–4.33) and lower *T*-scores at the PA total spine (MD = 2.66, 95% CI = 2.11–3.21), lateral spine (MD = 1.40, 95% CI = 0.73–2.06), and total hip (MD = 1.86, 95% CI = 1.44–2.28). The GIO group also had significantly lower *T*-scores at the PA total spine (MD = 1.67, 95% CI = 0.85–2.48) and significantly higher FRAX hip scores (MD = 1.67, 95% CI = 0.42–2.43) compared to controls. Both the GIO and primary osteoporosis groups had significantly higher FRAX major osteoporotic fracture scores than controls (MD = 3.42, 95% CI = 0.89–5.95 and MD = 1.96, 95% CI = 0.13–2.80, resp.).

### 3.2. Differences in Mean Areal srBMD between Groups

#### 3.2.1. Sagittal Subregions

**Figure 2:**
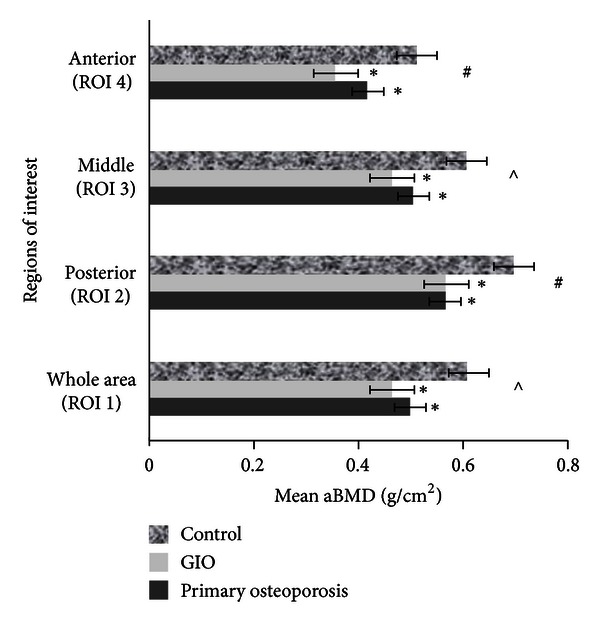
ANOVA 1, mean areal BMD for sagittal regions of interest between groups, adjusted for ROI 1 area. Error bars are SEM. *Significantly different to controls (*P* < 0.0001). ^#^Significantly different compared to all other ROIs. ^*∧*^Significantly different to ROIs 2 and 4.

A main effect for subregion was observed (*F* = 4.29, *P* < 0.01), with mean adjusted aBMD in all subregions being significantly different from each other (*P* < 0.0001). All analyses were adjusted for ROI 1 area, to account for differences in vertebral size between groups. One exception was observed for the whole vertebral body (ROI 1) mean adjusted aBMD versus middle subregional (ROI 3) mean adjusted aBMD (*P* = 1.00). The lowest adjusted mean (±SD) aBMD was observed in the anterior subregion (0.411 ± 0.126 g/cm^2^) (ROI 4) and the highest in the posterior subregion (0.583 ± 0.137 g/cm^2^) (ROI 2), and importantly, these adjusted mean srBMD values varied significantly from the adjusted mean srBMD of the whole vertebral body (Figure 2). A main effect for group (*F* = 15.75, *P* < 0.0001) was observed, reflecting lower adjusted mean srBMD in both the GIO and primary osteoporosis groups compared to controls (MD = 0.14 g/cm^2^, 95% CI = 0.05–0.24 and MD = 0.12 g/cm^2^, 95% CI = 0.05–0.18, resp.) (Figure 2). No significant difference was observed between the GIO and primary osteoporosis groups. 

No significant group × subregion interaction main effect was observed (*F* = 1.44, *P* = 0.169); however, adjusted mean (±SD) aBMD in the anterior subregion (ROI 4) was lower in the GIO group (0.355 ± 0.148 g/cm^2^) compared to the primary osteoporosis group (0.416 ± 0.079 g/cm^2^, MD = 0.06 g/cm^2^, 95% CI = 0.031–0.09) (*P* = 0.017), a result not observed at any other subregion. This finding represented an effect size (Cohen's *d*) of *d* = 0.51.

#### 3.2.2. Transverse Subregions

**Figure 3:**
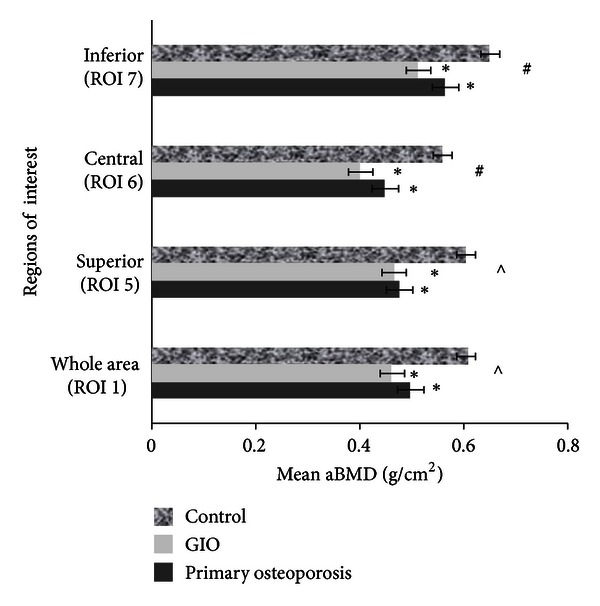
ANOVA 2, mean areal BMD for transverse regions of interest between groups, adjusted for ROI 1 area. Error bars are SEM. *Significantly different to controls (*P* < 0.0001). ^#^Significantly different compared to all other ROIs. ^*∧*^Significantly different to ROIs 6 and 7.

A main effect for subregion was observed (*F* = 2.80, *P* = 0.04), with mean adjusted aBMD in all subregions being significantly different from each other (*P* < 0.0001), with the exception of the whole vertebral body (ROI 1) adjusted mean aBMD versus superior subregional (ROI 5) adjusted mean aBMD (*P* = 1.00). The lowest adjusted aBMD was observed in the central subregion (0.451 ± 0.133 g/cm^2^) (ROI 6) and the highest in the inferior subregion (0.559 ± 0.122 g/cm^2^) (ROI 7), and importantly, these adjusted mean srBMD varied significantly from the adjusted mean srBMD of the whole vertebral body (Figure 3). A significant main effect for group (*F* = 15.96, *P* < 0.0001) was observed, with lower adjusted mean srBMD in both the primary osteoporosis and GIO groups compared to controls (MD = 0.108 g/cm^2^, 95% CI = 0.05–0.17 and MD = 0.14 g/cm^2^, 95% CI = 0.06–0.23, resp.) (Figure 3). No significant group × subregion interaction was observed (*P* = 0.88). 

### 3.3. Ratios of srBMD

Table 2 displays srBMD ratio comparisons between groups. Comparing subregions to the whole vertebral area, the GIO group demonstrated a significantly higher ratio for ROI 2 : ROI 1 (1.27) when compared with the primary osteoporosis (1.13; MD = 0.13, 95% CI = 0.04–0.23) and control groups (1.16; MD = 0.12, 95% CI = 0.01–0.20) (*P* < 0.05) and a significantly lower ratio for ROI 4 : ROI 1 (0.72) when compared to the primary osteoporosis (0.84; MD = 0.13, 95% CI = 0.02–0.23) and control (0.83; MD = 0.12, 95% CI = 0.01–0.22) groups (*P* < 0.05). Comparing between subregions, the GIO group demonstrated a significantly lower ratio for ROI 4 : ROI 2 (0.59), compared to the primary osteoporosis (0.76; MD = 0.17, 95% CI = 0.03–0.31) and control (0.73; MD = 0.14, 95% CI = 0.00–0.27) groups (*P* < 0.05). No significant differences in srBMD ratios were observed between the primary osteoporosis and control groups for any ratio.

## 4. Discussion

### 4.1. Main Finding

Primarily, this study has shown that DXA can identify differences in srBMD within a vertebra, as displayed by the significant main effect for subregion (*P* < 0.05), consistent with earlier data [38, 42, 43]. Secondly, it was found that the GIO group demonstrated significantly lower results for the anterior subregion : whole vertebra ratio (ROI 4 : ROI 1) and the anterior subregion : posterior subregion ratio (ROI 4 : ROI 2) when compared to both the primary osteoporosis and control groups. These data may help explain why vertebral fractures occur at relatively higher PA aBMD values than what is seen in typical primary osteoporosis, since the intravertebral distribution of bone appears to be abnormal in glucocorticoid-treated patients but not in patients with primary osteoporosis. The findings indicate that the anterior subregion may be of particular interest in a GIO population for assessing individuals' risk of vertebral fracture and that the ratio technique may prove useful in a clinical setting as a means of determining bone distribution without needing to adjust for vertebral size.

### 4.2. Sagittal Subregional Intravertebral aBMD Profile (ROIs 2–4)

Main effects for both subregion and group were observed, demonstrating that this study, along with the earlier pilot work [56], has been able to show differences in subregional aBMD *in vivo*. The lowest mean srBMD was observed in the anterior subregion (ROI 4), a result consistent with previous *ex vivo* histomorphometry, QCT, and DXA studies [36–40, 42, 43] and the highest was in the posterior subregion (ROI 2). This distribution pattern is probably explained by a combination of a higher cortical bone component in the posterior subregion compared to the anterior subregion and a result of changes in vertebral loading profiles, in particular intervertebral disc degeneration and changes in lumbar spine posture [21, 57]. When disc degeneration occurs, force loading increases on the neural arch, hence decreasing loading on the anterior region and causing regional demineralisation [57–59]. Although no significant group × subregion interaction effect was observed, we detected significantly lower srBMD in the anterior subregion (ROI 4) for the GIO group compared to the primary osteoporosis group. Despite a significant finding with a medium effect size, this result should be considered in the context of a relatively small sample size and disproportionate sex distribution. Nonetheless, this result occurred despite the larger proportion of male patients in the GIO group which intuitively would have increased the mean aBMD in the GIO group, relative to a female-dominated GIO group. If all confounding factors were removed and sample sizes increased and balanced between the groups, this difference in aBMD may have been even more marked. We observed a power of 44% for this two-tailed analysis, suggesting that with a larger sample size, in the order of 60 per group, a power of 80% could be achieved. 

### 4.3. Transverse Subregional Intravertebral aBMD Profile (ROIs 5–7)

As with the sagittal subregions, main effects were observed for both subregion and group. The central subregion (ROI 6) exhibited the lowest aBMD, a result consistent with previous *ex vivo* studies [36–40, 42, 43]. The highest observed aBMD was in the inferior subregion (ROI 7) and may be explained due to this region's proximity to the chondral endplate, an area rich in cortical bone [60]. There was no significant subregion × group interaction observed nor were there any significant differences in supero-inferior ratios. This was not consistent with previous findings suggesting lower bone content at the central subregion. 

### 4.4. Ratios

While it is possible to compare srBMD between groups, caution is required due to variability in subregional bone geometry. The comparison of ratios is therefore a feasible approach to account for differences in vertebral size and may reduce gender effects caused by differences in bone size. Ratios also provide information on the distribution of bone within the subregions; hence the lower observed ROI 4 : ROI 2 and ROI 4 : ROI 1 ratios suggest less bone is distributed into the anterior subregion compared to the posterior subregion and vertebral body as a whole. This may suggest that the anterior subregion is at an increased risk of fracture (particularly anterior wedge fracture) within individuals exposed to glucocorticoid therapies, compared to other subregions, whilst the posterior subregion appears to be less affected. 

### 4.5. Strengths and Limitations

Strengths of this study include the use of the widely employed Genant semiquantitative approach [46] along with vertebral height reference data [53] for fracture diagnosis and the use of our group's established, reliable, and validated subregional analysis protocol [41–43, 45, 54]. The relatively small sample in the GIO group led to a sex imbalance between groups. Despite the GIO group being predominantly male compared to the other groups which were predominantly female, statistically and clinically-significant deficits were found in the glucocorticoid group. Nonetheless, we cannot exclude the possibility that differences in intravertebral bone distribution were related to sex differences between groups. The use of a cross-sectional study design does not allow conclusions about causal relationships and thus future longitudinal work would be beneficial in strengthening this study's findings. 

### 4.6. Future Directions

The use of intravertebral aBMD ratios to indicate abnormalities in bone distribution has the potential for application in a clinical setting, especially for men, where limited lateral reference data are available. The subregional approach may enhance the ability to monitor the effects of bone-active medications on bone architecture. Intervention studies would lend further support to this application of the technique.

## 5. Conclusions

A robust connection exists between aBMD and vertebral strength, hence indicating the important role of BMD in reflecting fracture risk. This study has shown that a lateral subregional approach to aBMD measurement provides greater information than standard PA-projection DXA about differences in aBMD between individuals with GIO and individuals with primary osteoporosis. In particular, glucocorticoid-treated patients had abnormal intravertebral distribution of bone, with relatively low aBMD in the anterior subregion. Glucocorticoid-treated individuals at high risk of sustaining vertebral fractures may be better identified with the clinical application of this technique.

## Figures and Tables

**Table 1:** Descriptive measures for each group and pooled data, expressed as the mean (SD) for age, height, mass, and BMI and as the mean (95% CI) for FRAX data, *T*-scores, and L3 area.

Descriptive measure	Group			*Pooled*
	Primary osteoporosis	GIO	Control	
*N* (% female)	43 (95.3)	13 (30.8)	48 (83.3)	*104 (81.7) *
Age (years)	61.1 (5.0)	63.7 (7.8)	61.5 (5.6)	*62.1 (6.1) *
Height (cm)	163.1 (7.6)	169.1 (8.7)	166.8 (6.5)	*166.3 (7.6) *
Mass (kg)	61.8 (10.0)^b,c^	75.6 (16.1)^a^	71.9 (8.8)	*69.8 (11.6) *
BMI (kg/m^2^)	23.2 (3.0)^b,c^	26.2 (3.7)^a^	25.8 (2.8)	*25.1 (3.2) *
FRAX major osteoporotic (%)	4.5 (3.7 to 5.4)^c^	6.0 (3.3 to 8.7)^c^	2.6 (2.0 to 3.2)	*4.4 (3.0 to 5.8) *
FRAX hip (%)	1.1 (0.8 to 1.4)	2.1 (0.8 to 3.4)^c^	0.4 (0.1 to 0.7)	*1.2 (0.6 to 1.8) *
*T*-score PA spine (L1–4)	−2.6 (−2.9 to −2.4)^b,c^	−1.6 (−2.4 to −0.9)^a,c^	0.0 (−0.2 to 0.3)	*−1.4 (−1.8 to −1.0) *
*T*-score total hip	−2.0 (−2.2 to −1.8)^b,c^	−1.3 (−1.9 to −0.8)^a,c^	−0.2 (−0.4 to 0.1)	*−1.8 (−1.5 to −0.8) *
L3 area (ROI 1) (cm^2^)	8.8 (8.1 to 9.5)^b^	11.1 (10.1 to 12.0)^a^	9.9 (9.1 to 10.0)	*9.9 (9.1 to 10.5) *

^a^Significant difference compared to the primary osteoporosis group (*P* < 0.01); ^b^significant difference compared to the GIO group (*P* < 0.01); ^c^significant difference compared to control group (*P* < 0.01).

**Table 2:** Mean (95% CI) ratio values of srBMD for all groups.

Ratio for subregions	Primary osteoporosis	GIO	Control
Posterior : whole (2 : 1)	1.13 (1.09–1.17)	**1.27 (1.15–1.38)** ^ a^	1.16 (1.12–1.19)
Middle : whole (3 : 1)	1.01 (0.99–1.03)	1.01 (0.92–1.10)	1.00 (0.98–1.01)
Anterior : whole (4 : 1)	0.84 (0.81–0.88)	**0.72 (0.58–0.86)** ^ a^	0.83 (0.80–0.86)
Superior : whole (5 : 1)	0.95 (0.93–0.98)	1.01 (0.94–1.10)	1.00 (0.96–1.04)
Central : whole (6 : 1)	0.90 (0.87–0.92)	0.85 (0.78–0.92)	0.92 (0.89–0.95)
Inferior : whole (7 : 1)	1.14 (1.12–1.18)	1.13 (1.02–1.24)	1.08 (1.05–1.11)
Middle : posterior (3 : 2)	0.90 (0.86–0.95)	0.81 (0.71–0.91)	0.87 (0.84–0.90)
Anterior : posterior (4 : 2)	0.76 (0.70–0.82)	**0.59 (0.45–0.74)** ^ a^	0.73 (0.69–0.77)
Anterior : middle (4 : 3)	0.84 (0.80–0.88)	0.75 (0.56–0.93)	0.84 (0.80–0.88)
Central : superior (6 : 5)	0.95 (0.91–1.00)	0.85 (0.75–0.95)	0.94 (0.89–0.99)
Superior : inferior (5 : 7)	0.85 (0.80–0.90)	0.93 (0.77–1.09)	0.95 (0.88–1.01)
Central : inferior (6 : 7)	0.80 (0.76–0.84)	0.78 (0.65–0.91)	0.86 (0.81–0.92)

^a^Significant difference compared to both primary osteoporosis and control groups (*P* < 0.05).
